# Supplementary material for: Impact of a 24-Week Workplace Physical Activity Program on Oxidative Stress Markers, Metabolic Health, and Physical Fitness: A Pilot Study in a Real-World Academic Setting
Source: J Funct Morphol Kinesiol. 2025 Sep 12;10(3):348. doi: 10.3390/jfmk10030348 (PMC12452330; doi:10.3390/jfmk10030348)
Supplement: Supplementary file 1 [file jfmk-10-00348-s001.zip › jfmk-3822525-supplementary.pdf]

Table S1 – Intervention scheme across the 24 weeks.

**PF components[1]**	**Weeks 1-2**	**Weeks 3-4**	**Weeks 5-6**	**Weeks 7-8**	**Weeks 9-10**	**Weeks 11-12**	**Weeks 13-14**	**Weeks 15-16**	**Weeks 17-18**	**Weeks 19-20**	**Weeks 21-22**	**Weeks 23-24**
CRF	3	2	2	3	2	2	3	2	2	3	2	2
MS	2	3	2	2	3	2	2	3	2	2	3	2
ME	2	2	3	2	2	3	2	2	3	2	2	3
FLEX/BAL	1	1	1	1	1	1	1	1	1	1	1	1
Total station N°	8	8	8	8	8	8	8	8	8	8	8	8

[1] Numbers indicate the exercise stations for each Physical Fitness component

PF: Physical Fitness

CRF: Cardio-Respiratory Fitness

MS: Muscular Strength

ME: Muscular Endurance

FLEX/BAL: Flexibility and Balance

Figure S1: Implemented circuit in week 1 and 2 of the WPAP.

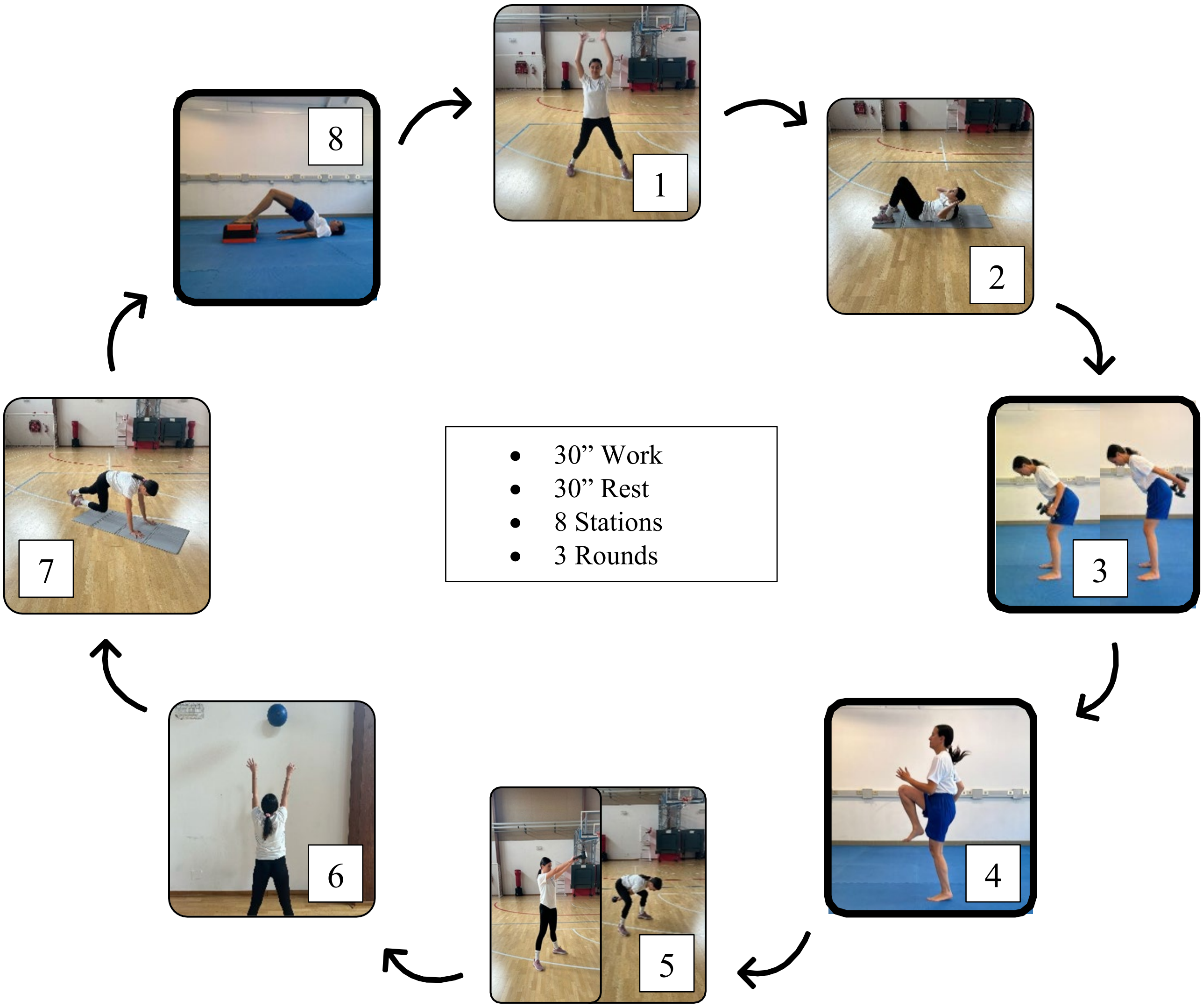

Table S2: Examples of the different stations of Circuit Training.

Stations	Circuit1[1]	Circuit2	Circuit3
1	Jumping jack	Step touch	Burpees
2	Classic crunch	Bodyweight squat	Sit ups
3	Triceps kickback	Dead Bug	French press
4	High Knee Skip	Side lunges	Split jumps
5	Kettlebell Swing	Running in place	Biceps curl
6	Wall Ball	Shoulder touch	Walking lunges
7	Mountain climber	Russian Twist	Shuttle run
8	Hip-Thrust	Swiss Ball Plank	Single-leg Romanian deadlift

[1]Circuit1 is referred to Figure S1 and has been implemented in weeks 1 and 2 of the WPAP.

Table S3: list of the equipment used for the WPAP.

	Equipment
1	Gym mats
2	Dumbbells (2 kg, 3 kg, 5 kg, 7 kg, 8 kg, 10 kg)
3	Resistance bands
4	Kettlebells (6 kg, 8 kg, 10 kg, 12 kg, 15 kg, 20 kg)
5	Wooden sticks
6	Medicine balls (1 kg, 2 kg, 4 kg, 5 kg)
7	Tennis, volley, basket balls
8	Chairs
9	Benches
10	Cones
11	TRX
12	Punching bags
13	Sandbags (5 kg, 10 kg)
14	Plyo boxes
15	Steppers
16	Jump ropes
17	Bosu balls
18	Swiss balls

Figure S2: Mobility training session examples (15”–30” or 12–20 repetitions per exercise, with 2–4 sets)

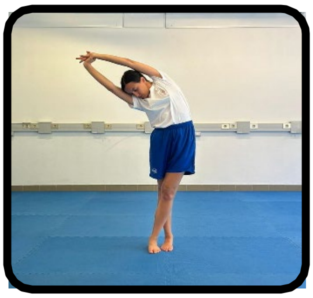

1. Standing Side Bend

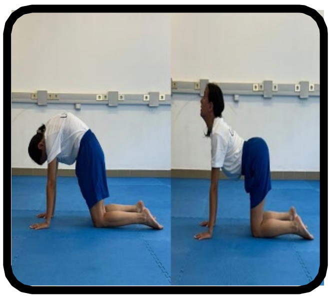

2. Cat – Cow

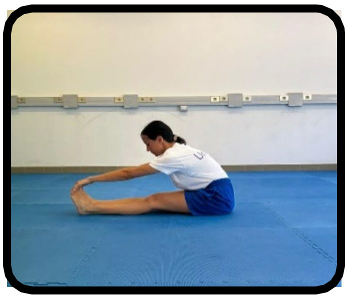

3. Seated forward bend

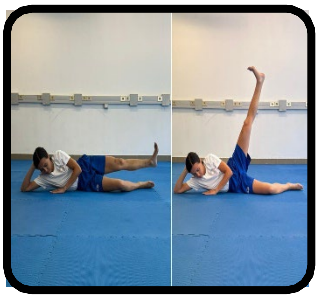

4. Side-lying Leg Lift

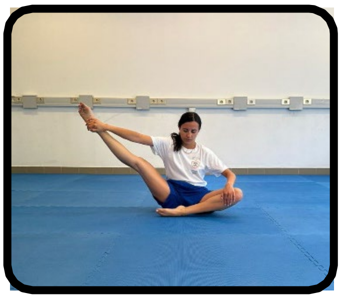

5. Seated leg stretch

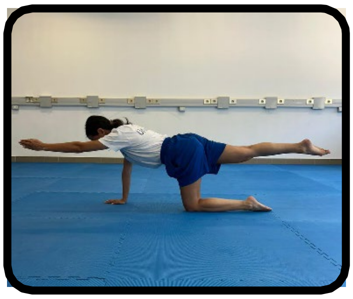

6. Bird Dog

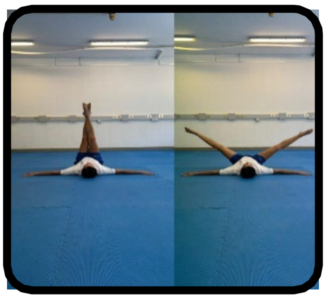

7. Leg scissors

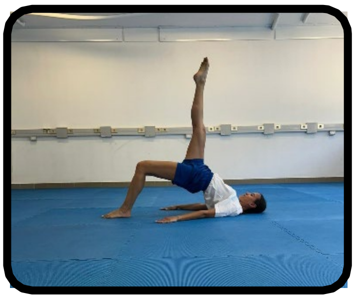

8. Single-leg Glute bridge

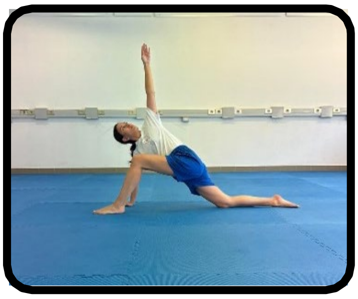

9. Posterior Chain Stretch

Figure S3: Participant flowchart.

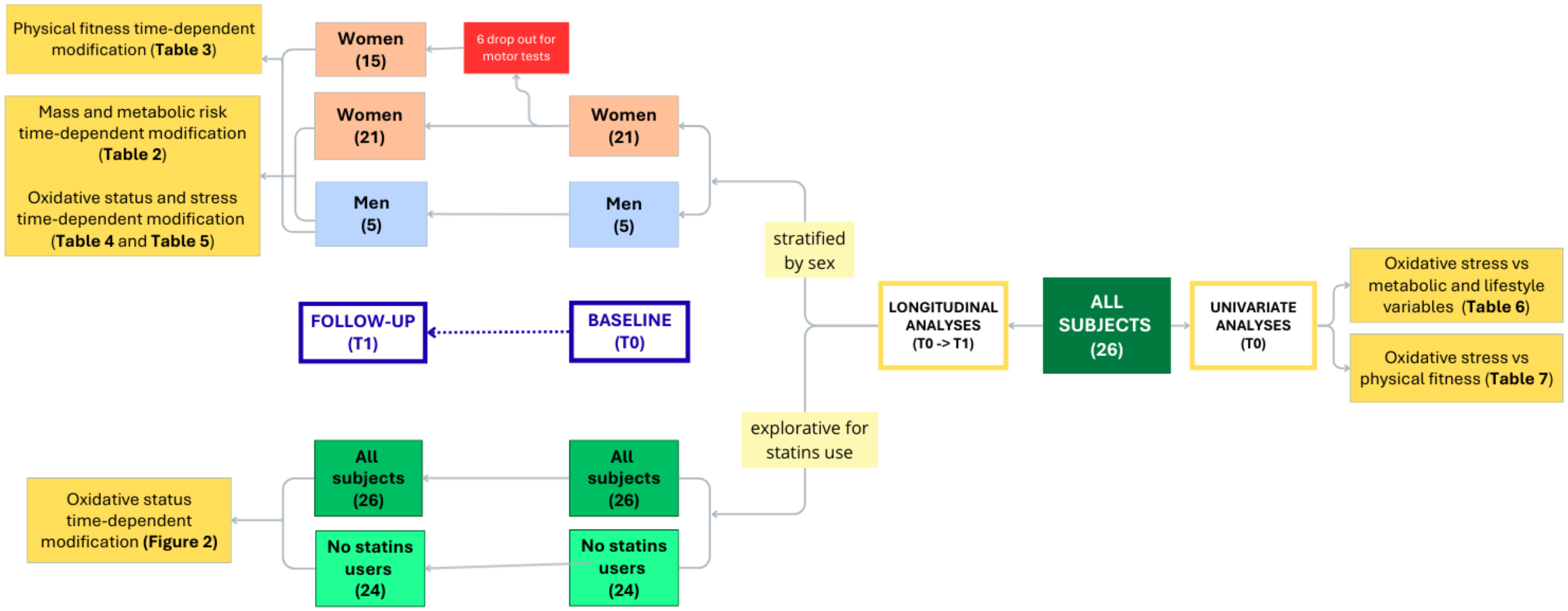

Table S4: BAP and d-ROMs levels

	**BAP Levels**					
	**Optimal state**	**Borderline**	**Slight deficit**	**Deficit**	**Severe deficit**	**Extreme deficit**
	(>2200 µmol/L)	(2200-2000 µmol/L)	(2000-1800 µmol/L)	(1800-1600 µmol/L)	(1600-1400 µmol/L)	(<1400 µmol/L)
**d-ROMs levels**						
**Below normal** (<250 U-CARR)						
**Normal** (250-300 U-CARR)						
**Borderline** (301-320 U-CARR)						
**Low O.S.** (321-340 U-CARR)						
**Medium O.S.** (341-400 U-CARR)						
**High O.S.** (401-500 U-CARR)						
**Very high O.S.** (≥500 U-CARR)						

Translated in Italian from original, by Iorio et al. [41] (O.S. = Oxidative stress)

Case 1	**d-ROMs ↓ BAP ↓**	**Absolute hyporeactivity**
Case 2	**d-ROMs ↓ BAP ~**	**Relative hyporeactivity**
Case 3	**d-ROMs ~ BAP ~**	**Optimal oxidative balance**
Case 4	**d-ROMs ~ BAP ↓**	**Relative oxdiative stress**
Case 5	**d-ROMs ↑ BAP ~**	**Potential oxidative stress**
Case 6	**d-ROMs ↑ BAP↓**	**Absolute oxidative stress**

Table S5: Paired effect sizes with 95% CIs for all pre-post changes.

	Oxidative status variables by time (means±dev.st.)		Absolute change t1-t0 (Δ)				Wilcoxon signed-rank test (matched pairs)			Effetc size				pwcorrelation pre-post		Post-hoc achieved power (A.R.E. method)
	t0	t1	aritm. mean	st.dev	C.I. 95%		Z	N	p [(a)]	**Wilcoxon's r [(b)]**		**Cohen's d [(c)]**		r	sign.	(1-β err prob.)
**d-ROMs - F**	383.2±79.3	426.8±87.3	43,6	54,9	18,6	68,6	-2,816	21	0,0033	-0,435	medium	0,725	medium	0,7866	p<0,001	0,868
**d-ROMs - M**	338.4±38.9	388.0±66.4	49,6	42,2	-2,8	102,0	-1,753	5	0,1250	-0,554	large	1,175	large	0,8017	n.s.	0,484
**BAP-F**	2513.8±624.6	2640.4±488.2	126,6	935,0	-299,0	552,2	-0,434	21	0,6827	-0,067	small	0,135	small	-0,4028	n.s.	0,089
**BAP-M**	2578.2±943.6	2789.2±379.6	211,0	1052,4	-1095,7	1517,7	-0,405	5	0,8125	-0,128	small	0,200	small	-0,1020	n.s.	0,063
**BAP/d-ROMs - F**	7.0±2.8	6.4±1.6	-0,6	2,8	-1,9	0,7	0,678	21	0,5168	0,105	small	0,214	small	0,2871	n.s.	0,065
**BAP/d-ROMs - M**	7.6±2.7	7.4±1.5	-0,2	2,8	-3,7	3,3	0,135	5	1,0000	0,043	small	0,072	small	0,2148	n.s.	0,052

[(a)] exact p value computation since the small sample size

[(b)] Wilcoxon's r cut-offs
small = 0,10
medium = 0,30
high = 0,50

[(c)] Cohen's d cut-offs
small = 0,2
medium = 0,5
high = 0,8
